# Milk Fat Globules Hamper Adhesion of Enterohemorrhagic *Escherichia coli* to Enterocytes: *In Vitro* and *in Vivo* Evidence

**DOI:** 10.3389/fmicb.2018.00947

**Published:** 2018-05-15

**Authors:** Thomas Douëllou, Wessam Galia, Stéphane Kerangart, Thierry Marchal, Nadège Milhau, Renaud Bastien, Marion Bouvier, Samuel Buff, Marie-Christine Montel, Delphine Sergentet-Thevenot

**Affiliations:** ^1^Institut National de Recherche Agronomique, Unité de Recherches Fromagères, Aurillac, France; ^2^Université de Lyon, Research Group “Bacterial Opportunistic Pathogens and Environment”, UMR5557 Ecologie Microbienne Lyon, Université Lyon 1, CNRS, VetAgro Sup, Marcy-l’Étoile, France; ^3^UPSP ICE 2011.03.101 & CRB ANIM (ANR11.INBS.0003), Université de Lyon, VetAgro Sup, Marcy-l’Étoile, France; ^4^Department of Collective Behaviour, Max Planck Institute for Ornithology, Konstanz, Germany; ^5^Department of Biology, University of Konstanz, Konstanz, Germany; ^6^Laboratoire d’Études des Microorganismes Alimentaires Pathogènes – French National Reference Laboratory for Escherichia coli Including Shiga Toxin Producing E. coli, Université de Lyon, VetAgro Sup Campus Vétérinaire, Marcy-l’Étoile, France

**Keywords:** EHEC, milk fat globules, inhibition of colonization, *in vitro*, *in vivo*

## Abstract

Enterohemorrhagic *Escherichia coli* (EHEC; *E. coli*) are food-borne agents associated with gastroenteritis, enterocolitis, bloody diarrhea and the hemolytic-uremic syndrome (HUS). Bovine milk glycans have been shown to contain oligosaccharides which are similar to host epithelial cell receptors and can therefore prevent bacterial adhesion. This study aimed to describe interactions between EHEC O157:H7 EDL933 and O26:H11 21765 and milk fat globules (MFGs) in raw milk and raw milk cheese, and the impact of MFGs on EHEC strains adhesion to the intestinal tract *in vitro* and *in vivo*. Both EHEC serotypes clearly associated with native bovine MFGs and significantly limited their adhesion to a co-culture of intestinal cells. The presence of MFGs in raw milk cheese had two effects on the adhesion of both EHEC serotypes to the intestinal tracts of streptomycin-treated mice. First, it delayed and reduced EHEC excretion in mouse feces for both strains. Second, the prime implantation site for both EHEC strains was 6 cm more proximal in the intestinal tracts of mice fed with contaminated cheese containing less than 5% of fat than in those fed with contaminated cheese containing 40% of fat. Feeding mice with 40% fat cheese reduced the intestinal surface contaminated with EHEC and may therefore decrease severity of illness.

## Introduction

Enterohemorrhagic *Escherichia coli* (EHEC) are food-borne bacteria responsible of several outbreaks over the globe. EHEC are strongly associated with hemorrhagic colitis and hemolytic-uremic syndrome (HUS) that represent a serious public health concern. Numerous epidemic and isolated cases have been attributed to EHEC of the O157:H7 serotype. However, others serogroup of STEC, such as O26, O45, O103, O111, O121, and O145 have been also implicated in HC and HUS cases (EFSA and ECDC, 2015). Furthermore a STEC O104:H4 strain, which had both enteroaggregative and enterohemorrhagic properties was implicated in 2011 into a large outbreak in Germany and France (Buchholz et al., 2011). In 2014, 5955 confirmed cases of EHEC infections were reported by 25 European Union member states, a notification rate of 1.56 cases per 100,000 individuals (EFSA and ECDC, 2015). These infections led to seven deaths, corresponding to a European overall fatality rate due to EHEC infection of 0.2% (EFSA and ECDC, 2015). In France, in 2013, 152 cases of HUS have been reported. The annual incidence of HUS in France is 1.2 per 100,000 children under 15 years. Human infection is mainly link to the ingestion of contaminated raw ground beef, dairy products, vegetables or water. Children and elderly people are the groups at highest risk of HUS. The infectious dose of EHEC that can cause outbreaks was estimated very low (from 5 to 50 viable cells) (Nataro and Kaper, 1998). Official monitoring conducted in 2009 evaluated the prevalence of Shiga toxin-producing *E. coli* (STEC; potential EHEC strains) in French raw milk cheeses at 0.9% (Loukiadis et al., 2012). Nevertheless raw milk cheeses are only a minor source of EHEC human infections [EFSA BIOHAZ Panel (EFSA Panel on Biological Hazards, 2015)].

Shiga toxins (Stxs) are the main virulence factors in EHEC, but although Stx is a necessary condition for virulence, other EHEC properties, including adherence to the intestinal surface and colonization of the gut, obviously contribute to the pathogenic process. To colonize the host and cause disease, EHEC have evolved means for adhering to the host cells and tissues (Ofek et al., 2003; Kaper et al., 2004). Adhesion is required so limit the elimination of the organisms by the intestinal flux. Bacteria expresses several adhesion factors on their surfaces for their adhesion. Some of its are curly fimbriae and lectins that interact with specific carbohydrates present on the host cell surfaces (Douellou et al., 2017). Most of the typical EHEC strains are defined by the presence of the *eae* gene (Croxen and Finlay, 2010). The gene encodes one of the proteins responsible for attachment and interaction between bacteria and epithelial cells, resulting in the formation of attaching-and-effacing (AE) lesions in human ileum and colon (Croxen and Finlay, 2010). Several other bacterial adhesins have been characterized for *E. coli* (Klemm et al., 2010). Interestingly, several natural and synthesized food components (oligosaccharides) have been shown to act as efficient inhibitors of intestinal pathogen adherence (Douellou et al., 2017).

Milk and cheese contain a large variety of components encoding molecules that offer functional health benefits, such as complex carbohydrates. Indeed, bovine milk glycans have been shown to prevent bacterial adherence, since they contain oligosaccharides similar to host epithelial cell receptors and can therefore act as efficient decoys (Bao et al., 2007; Douellou et al., 2017). MUC1 is the most efficient milk fat globule membrane (MFGM)-associated glycoprotein for inhibiting bacterial adhesion (Wang et al., 2001; Sprong et al., 2002), even if other MFGM-glycoconjugate fractions are also implicated (Struijs et al., 2013). Previous work on MFGM inhibition of pathogens adhesion, including *E. coli* and *Salmonella*, demonstrated the effectiveness of these glycoconjugates to limit bacterial colonization (Guri et al., 2012; Ross et al., 2016). However, to the best of our knowledge, the impact of MFG on EHEC adhesion *in vivo* has not been investigated.

This study aims (i) to evaluate the association of EHEC O157:H7 EDL933 and O26:H11 21765 with milk fat globules (ii) to assess the ability of milk fat globules to prevent adhesion of EHEC to digestive tract cells. EHEC association with MFGs was studied by contaminated milk cream separation assays and microscope observation of MFG/EHEC in raw milk. Anti-adhesion capacity was studied (a) by examining EHEC adhesion to a co-culture of intestinal cells lines *in vitro* and (b) by examining EHEC adhesion to the intestinal tracts of mice fed with EHEC-contaminated cheese.

## Materials and Methods

### EHEC Strains and Culture Conditions

*Escherichia coli* O26:H11 strain 21765 (Galia et al., 2015) and *E. coli* O157:H7 strain EDL933 (Perna et al., 1998) were studied. Both strains were sequenced and genome sequence and annotation are available in international databases NCBI (RefSeq *E. coli* O157:H7 strain EDL933: NZ_CP008957.1; RefSeq *E. coli* O26:H11 strain 21765^1^; contig accession numbers CDLB01000001 to CDLB01000102). For the studies of EHEC colonization mouse intestinal tract, a spontaneous streptomycin-resistant (Str^r^) mutant was derived from each of the clinical EHEC isolates (named 21765 SR225 and EDL933 SR225, respectively). Briefly, wild type strains were first revived from frozen stock by incubation 24 h at 37°C in 9 mL brain heart infusion (BHI, bioMérieux, Marcy-l’Étoile, France). Each one was isolated on plate-count agar (PCA, bioMérieux, Marcy-l’Étoile, France) and incubated at 37°C for 24 h. BHI containing 3.52–225 μg/mL of streptomycin sulfate were prepared by twofold serial dilution with sterile BHI. One colony of each strain was picked and cultured on streptomycin sulfate supplemented BHI overnight at 37°C. The highest concentration of streptomycin resistant strain was used to re-inoculate BHI containing higher antibiotic concentrations until obtaining a spontaneous mutant resistant to 225 μg/mL of streptomycin. All the Str^r^ derivative strains had similar growth rates in BHI to those of the parental strain (See Supplementary Figure S2).

Fresh cultures derived from the frozen stock were used for each experiment. Mutants were revived in BHi supplemented with 100 μg/mL of streptomycin sulfate in order to maintain the selection pressure.

### Localization of EHEC Strains in Milk Phases

#### Cream Separation Assay

One hundred milliliters of raw milk contaminated with approx. 7 Log_10_, 6 Log_10_, 5 Log_10_, 4 Log_10_, or 3 Log_10_ colony forming unit (CFU)/ml of EHEC (O26:H11 strain 21765 or O157:H7 strain EDL933) were placed into a 100 mL graduated cylinder. The fat was labeled with 1 μg/mL Nile red (Sigma, St. Louis, MO, United States). The fat was allowed to settle passively at 4°C for 24 h.

Enterohemorrhagic *Escherichia coli* were counted in “skimmed” phase and in the “fat” phase on Brilliance *E. coli* medium (ECB; Oxoid, Dardilly, France). Results were expressed as a percentage of EHEC concentration in each phase with respect to the initial concentration introduced into the raw milk. Controls were performed in ultra high temperature (UHT) treated skimmed milk. Microbial population of raw milk was also counted on PCA medium (48 h, 37°C) in these different fractions of uncontaminated raw milk.

#### Milk Sample Preparation for Epifluorescence Microscopy Observation of EHEC Localization

Five hundred microliters of milk contaminated with O26:H11 strain 21765 or O157:H7 strain EDL933 as described in Section “Cream Separation Assay” were used for microscopic observations. Fluorescein-conjugated IgM anti-O26 [bioMerieux, Marcy-l’Étoile, France; Ratio Immuno-Globulin M (IgM): Fluorescein IsoThioCyanate (FITC) = 1:27] and either fluorescein-conjugated O157 specific recombinant phage protein (bioMerieux; Ratio = 1:8.9) or fluorescein-conjugated IgM anti-O157 (bioMerieux, Marcy-l’Étoile, France; Ratio IgM-FITC = 1:30) were added to contaminated milk at a final concentration of 5 μg/mL for each dye in order to specifically label EHEC O26:H11 strain 21765 and EHEC O157:H7 strain EDL933, respectively. Samples were incubated for 30 min at 4°C in the dark. Five microliters of sample were mounted between slide and cover slip. Suspensions were examined using an inverted microscope (IX-71; Olympus, Rungis, France) equipped for epifluorescence observations. Use of a conventional fluorescein long-pass filter coupled with direct light allowed simultaneous visualization of dyed EHEC strains and MFGs. Milk samples were photographed with a DP70 camera using a DP Controller, and were imported into DP Manager software (Olympus) as TIFF or JPEG files. Micrographs were processed using AxioVision Release Version 4.8 (Carl Zeiss, Oberkochen, Germany).

### Effect of MFGs on EHEC Adhesion to Intestinal Cells Lines Co-cultured *in Vitro*

#### Caco-2 Culture

The Caco-2 cell line was initially obtained from the American Tissue Culture Collection (ATCC No. HTB37). The cells were used at passages 38–45. They were cultured in Dulbecco’s Modified Eagle Medium (DMEM) supplemented with 10% fetal bovine serum, 1% nonessential amino acids, 100 U/mL penicillin and 100 U/mL streptomycin, at 37°C in a humidity-controlled 5% CO_2_ cell culture incubator. Cells were sub-cultured once a week with trypsin–Ethylenediaminetetraacetic acid (EDTA; 0.25%, 0.53 mM) and seeded at a density of 4 × 10^5^ cells per 75 cm^2^ flask. All cell culture media were purchased from Gibco BRL, Grand Island, United States or Life Technologies, Paisley, United Kingdom; other cell culture materials were obtained through VWR (VWR, Radnor, PA, United States) and Fisher Scientific, Ann Arbor, MI, United States. Medium was changed every other day.

#### HT29-MTX Culture

The mucus secreting HT29-MTX cells were kindly provided by Dr. A. Duval (INSERM UMR S 938, Paris, France) (Lesuffleur et al., 1990). HT29-MTX cells were maintained at 37°C, 5% CO_2_ in complete medium consisting of DMEM with 10% heat inactivated fetal bovine serum, 1% non-essential amino acids, and 100 U/ml penicillin and 100 μg/ml streptomycin. Cells were used at passages 8–12. They were sub-cultured once a week with trypsin-EDTA (0.25%, 0.53 mM) and seeded at a density of 4 × 10^5^ cells per 75 cm^2^ flask.

#### Co-cultures

Caco-2 and HT29-MTX cells were grown separately and predetermined numbers of each type of cell were mixed prior to seeding to yield a Caco-2 to HT29-MTX ratio of 9:1 (Hilgendorf et al., 2000). For adhesion assays, cells were seeded in cell culture inserts with polycarbonate membranes (pore size 0.4 μm, effective area 4.2 cm^2^) in six well culture plates at a density of 2.8 × 10^5^ cells per cm^2^ of insert, and were maintained under the same conditions as the stem cultures in DMEM modified medium described above. The culture medium was changed every 2 days and medium without antibiotics was used for the last two medium changes, which were done on consecutive days. Experiments were performed between 21 and 24 days post-seeding to reach confluent Caco-2 and HT29-MTX cells monolayer (Lesuffleur et al., 1993; Chen et al., 2010). The mucus production and epithelial integrity of Caco-2/HT-29 MTX co-cultures were checked in a negative control performed and analyzed by microscopy (Supplementary Figure S1).

#### Bacterial Adhesion Assay

Prior to the adhesion assay, the Caco-2-HT-29-MTX co-culture monolayer in each well was washed twice with 2 mL sterile PBS to remove residual culture medium. Bacterial cells from 12 h cultures grown at 37°C were centrifuged for 15 min at 3000 × *g* and 21°C. The pellets were washed once with PBS and re-suspended in milk. Then, skimmed raw milk (obtained by cream separation of raw milk for 48 h at 4°C), raw milk (∼3.8 g/kg fat), commercial UHT milk (3.6 g/kg fat) and commercial UHT skimmed milk (∼0.5 g/kg fat) were contaminated with EHEC O26:H11 strain 21765 or O157:H7 strain EDL933 to give bacterial cell to epithelial cell ratio of 10:1. Two milliliters of uncontaminated milk were added out of the insert and 2 ml of bacterial suspensions in milk (∼7.5 Log_10_ CFU/mL) were added to the tissue culture wells. The bacterial cells on the co-culture monolayers were incubated for 2 h at 37°C in 5% CO_2_ at 150 rpm. Following incubation, the unattached bacteria were removed by gently washing the co-culture monolayer with 2 ml of sterile PBS three times at 4°C. The co-culture monolayers were then scraped with 2 mL 0.1% (v/v) cold Triton X-100 (Sigma) per well on ice and passed three times through a 21 × g needle. One Milliliter of cells-bacteria suspension was used for serial 10-fold dilutions in PBS and plated onto chromogenic Brilliance *E. coli* agar. The plates were incubated at 37°C for 24 h. EHEC adhesion was expressed as the percentage of the number of bacteria adhering to 1 cm^2^ of cell culture compared to UHT commercial skimmed milk assays. Adhesion for UHT commercial skimmed milk was defined as 100% adhesion. Negative controls without bacteria and bacteria without cells were used throughout the experiment.

### Effect of Presence of Fat in Cheese on EHEC Adhesion to Mouse Intestinal Tract

### Cheese Making

For this experiment we used spontaneous strains, resistant to 225 μg/mL of streptomycin sulfate, of each EHEC strain (21765 SR225 and EDL933 SR225) (see section “EHEC Strains and Culture Conditions”). Each strain was revived as described above. EHEC biomass was measured by optical density at 600 nm (OD600). The EHEC strains were diluted in order to inoculate the raw milk before renneting at approx. 2 Log_10_ CFU ml^-1^.

Cheeses were made at the Unité de Recherches Fromagères (URF; INRA) in Aurillac, France, using an uncooked pressed cheese (UPC) method with a short ripening step (28 days) as described elsewhere (Miszczycha et al., 2013). Two models of cheeses were processed. For the first cheese model, raw milk was entirely skimmed using a creamer to obtain a fat concentration in cheese of approx. 5 g/kg. The other was a raw milk cheese containing 40 g/kg of fat, which is the natural concentration of fat in the UPC model. Eighteen cheeses were made: three cheeses per fat concentration (∼5 or 40 g/kg) and for each EHEC strain and 3 uncontaminated cheeses containing ∼5 and 40 g/kg of fat.

Samples for enumerating bacteria, fungi and EHEC were taken from milk and from cheese cores over time. Aseptically collected samples of 10 ml milk or 10 g cheese were diluted (1:10) in sterile Ringer solution and homogenized for 2 × 2 min using a stomacher (AES Laboratories). Ten-fold serial dilutions were performed with PBS and plated onto selective agar: EHEC strains, mesophilic and thermophilic bacterial populations, ripening bacteria, Gram negative bacteria, *Pseudomonas* and yeasts and molds were counted on selective agar as described in Supplementary Table S1. All counts were performed in triplicate (cf. Supplementary Figure S3).

For each trial, the raw milk was checked for the absence of *eae, stx* genes and *E. coli* O26:H11 or O157:H7.

#### Mouse Cheese Feeding and EHEC Inoculation

Streptomycin-treated mouse is a model of choice for studying intestinal colonization of EHEC strains (Luo et al., 2008) and has been extensively used for this purpose (Leatham et al., 2009). The number of persistent EHEC in streptomycin-treated mouse feces is a representation of their number in the large intestine (Leatham et al., 2009); where intestinal colonization is reduced, the level of EHEC in the feces is proportionately reduced (Leatham et al., 2009). Streptomycin sulfate was administrated to mice in their drinking water (5 g/liter) over the entire course of these experiments; this selectively removes facultative anaerobic and some strictly anaerobic bacteria (Hentges et al., 1984). However, the overall population of anaerobes in mouse feces following streptomycin treatment remain unchanged (Luo et al., 2008). We used fecal EHEC excretion to measure the relative intestinal colonizing abilities of EHEC O157:H7 strain EDL933 and EHEC O26:H11 strain 21765.

Eight sets of six to eight four-week-old male CD-1 mice (Harlan, Gannat, France) were used in this study, as described in **Table 1**. Mice were housed individually in cages with paper bedding.

**Table 1:** Composition of mouse sets used in this study and initial concentrations of O26:H11 strain 21765 and O157:H7 strain EDL933 in cheese matrices and in Enterohemorrhagic *Escherichia coli* (EHEC) suspension.

	Log_10_[O26:H11] CFU/g or mL	Log_10_[O157:H7] CFU/g or mL
∼0% fat cheese	4.28 (8)	4.09 (8)
40% fat cheese	4.41 (8)	4.14 (8)
EHEC suspension	5.69 (8)^a^	5.43 (8)^a^
∼0% fat cheese	–	5.48 (6)^a^
40% fat cheese	–	5.48 (6)^a^


**^a^Mice were pre-fed for 1 week with uncontaminated cheese containing ∼0% or 40% of fat before intra-gastric inoculation with 100 μL of EHEC suspension (Log [O157:H7] = 5.48 CFU/mL). Number shown in parentheses is the number of mice in each group*.*

During the 1-week adaptation, mice were fed with uncontaminated cheese containing ∼0 or 40% fat, depending on the pool to which they belonged (**Table 1**). Controls were fed with lab pet food (**Table 1**). Two days before the experiment, streptomycin sulfate (5 g/L) (Sigma) was given to mice in drinking water.

At D0, the streptomycin suspension was removed and the food was substituted by 2–3 g of EHEC-contaminated cheese (∼0 or 40% of fat, depending to the group). Mouse controls were intra-gastrically inoculated with 100 μL of an overnight suspension containing approx. 5.5 Log_10_ EHEC CFU mL^-1^ in order to contaminate them with approx. 4.5 Log EHEC SR225. After ingesting the contaminated cheese or the bacterial suspension, the mice were returned to normal diet with streptomycin-water.

One gram of feces was collected every 24 h for 7 days and mice were placed in clean cages at 24 h intervals, so that fecal samples were no older than 24 h. Seven days post feeding or post inoculation, the mice were euthanized and intestinal tracts were sampled from recto-anal junction to duodenum. One sample per EHEC strain and per cheese MFG concentration used for mice inoculation was used for “Swiss roll” epifluorescence microscopy observation; others were used to enumerate adhered EHEC.

Two experiments were performed to see whether MFGM glycoconjugates compete with EHEC for adhesion site or rather act as receptors for EHEC. In the first experiment, streptomycin treated mice were pre-fed with cheese containing ∼0 or 40% fat and then inoculated with a suspension of EHEC O157:H7 strain EDL933 SR225 by intra-gastric administration. In the second, streptomycin treated mice pre-fed with cheese containing ∼0 or 40% fat were fed with high- or low-fat cheese artificially contaminated with EHEC O157:H7 strain EDL933 SR225.

All animal protocols were approved by the Ethics Committee on Use and Care of Animals at the Veterinary campus at Marcy-l’Étoile, France, and by the national authorities (201507161750764 v1 (APAFIS#1185). All experiments were performed in accordance with relevant guidelines and regulations.

### Analysis of Mouse Samples

#### Real-Time PCR

Correlation between cycles to threshold (Ct) apparitions of O-antigens (O26-antigen or O157-antigen) or 16S rDNA genes and EHEC or bacterial concentration was determined using fresh cultures (BHI, 37°C) of O157:H7 strain EDL 933 SR225 and O26:H11 strain 21765 SR225 at end log phase (OD600 nm of approx. 0.8; See Supplementary Figure S4). Bacteria were counted by plating 10-fold serial dilutions in tryptone salt broth (bioMerieux) of fresh culture on PCA. Total gDNA was extracted using an EZ1 DNA Tissue Kit (Qiagen) and an EZ1 automatic DNA extractor in accordance with the manufacturer’s recommendations.

Calculation of equivalent cell number from real-time PCR data was obtained by amplifying serially diluted gDNA using a 10× dilution series to generate a standard curve according to the number of bacteria used for total gDNA extraction. Amplification of 16S rDNA gene of total gDNA was carried out in a total volume of 20 μl containing 10 μl of 2X SYBR Green PCR master mix, 300 nM of each primer as described by Park and Crowley (2006) and 5 μl of sample DNA diluted to 1/100 in nuclease-free water. SYBR Green real-time PCR was performed on a StepOne plus thermocycler (Thermo Fisher Scientific, Waltham, MA, United States) with a SYBR Green PCR master mix (Thermo Fisher Scientific), following the manufacturer’s instructions with slight modifications. Amplifications of O157- and O26-antigen were performed by TaqMan assay according to Perelle et al. (2004).

Mouse feces collected daily and intestinal tract sampled 7 days after feeding the mice on contaminated cheese were diluted in 9 mL of sterile PBS and disrupted and homogenized with 10000 rpm Ultra Turrax T25 (Janke & Kunkel, Germany) for 30 s. The fecal or organ suspensions were gently centrifuged for 1 min at 500 × *g* in order to form pellets. Total bacterial gDNAs were extracted from 1 mL of mouse feces or intestinal suspension using a BioRobot-EZ1 (Qiagen, Hilden, Germany), according to the manufacturer’s instructions. Total bacteria in feces or in intestinal layer were monitored by quantifying 16S rDNA gene as described elsewhere (Park and Crowley, 2006). Relative mouse intestinal colonization by EHEC was monitored daily by quantifying O-antigen in feces by rt-qPCR, using probes described by Perelle et al. (2004). EHEC colonization was expressed as equivalent bacteria per gram of feces. EHEC adhesion was expressed as equivalent bacteria per gram of organ. All rt-qPCR were performed at least in duplicate.

#### Histological “Swiss Roll” Preparation of Mouse Intestine

Swiss roll preparations of mouse intestine were prepared as described by Moolenbeek and Ruitenberg (1981) with slight modifications. One mouse per condition was analyzed. Briefly, after euthanasia, the section of the intestine from recto-anal junction to duodenum was taken from each mouse. Intestinal contents were eliminated and organ sections were washed three times with sterile physiologic water. The mesentery was removed. All samples were then placed for 56 h in 4% neutral buffered formalin in order to fix the organ and kill EHEC. Whole intestine sections were slit open with scissors from the caudal end. Organ section was carefully rinsed two times in formaldehyde. Samples were rolled around a wooden stick, mucosa on the outside. Preparations were placed into a tissue cassette that was immersed in 4% neutral buffered formalin and submitted to tissue processing and paraffin embedding followed by microtome cutting into 4 μm–thick sections. Paraffin sections were deparaffinized and rehydrated before antigen retrieval, performed in citrate buffer (pH 6.0). After rinsing, samples were blocked using UltraTek HRP anti-polyvalent kit (MM, Brignais, France) before incubation for 1 h in the dark with a primary monoclonal antibody against O-antigen conjugated with FITC (ratio 1:30 [IgM anti-O157], ratio 1:27 [IgM anti-O26]). After rinsing with PBS, samples were mounted using the Vectashield mounting medium for fluorescence with DAPI (Vector Laboratories, Burlingame, CA, United States), cover-slipped and scanned using an AxioScan Z1 (Zeiss).

#### Image Analysis

The position of each bacterium in the pictures was identified (*x*_b_, *y*_b_) using the Zen Blue software (Zeiss). Bacteria labeled with FITC and DAPI and attached to the intestinal layer when intestinal villi were entirely observable were counted. The contour of the basal lamina of the intestinal section was extracted by hand (∼300 points). The contour was then interpolated linearly between each two consecutive points so that the distance between points is of the order of some pixels using a MATLAB script. The curvilinear abscissa, s, along the organ was then directly computed so that the position of a point “i” is given by:

$$S_{i}=\overset{i}{\underset{j=1}{\Sigma }}\sqrt{{\left(x_{i}-x_{i-1}\right)}^{2}+{\left(y_{i}-y_{i-1}\right)}^{2},}S_{0}=0$$

**FIGURE 1:**
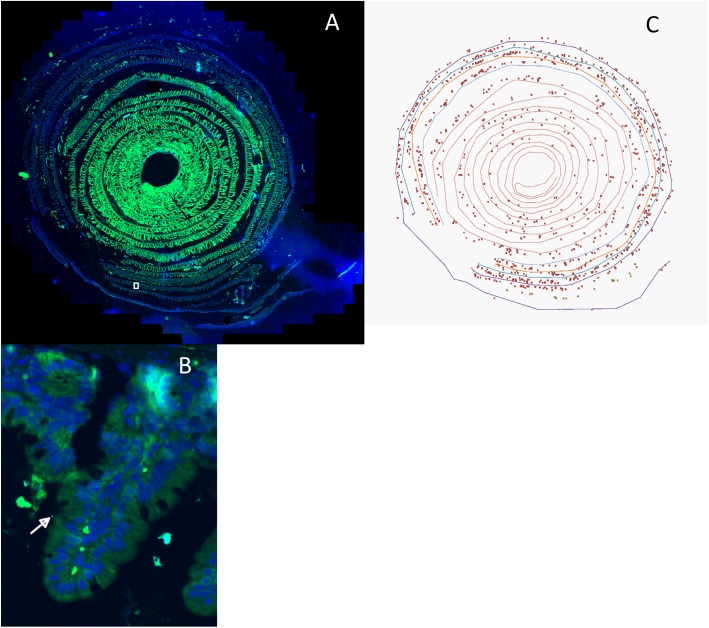
**(A)** Intestinal “Swiss roll” micrograph of a mouse fed with raw milk cheese contaminated with Enterohemorrhagic *Escherichia coli* (EHEC) O157:H7 strain EDL933 SR225. The intestinal sample was rolled from duodenum to recto-anal junction so that the anal junction was at the outer edge of the spiral. Intestinal cells were labeled using DAPI (Blue), EHEC cells were labeled using anti-O157 IgM coupled with FITC (Green). White square represents the area zoomed in **(B)**. **(B)** Micrograph of the zoomed area identified by the white square in **(A)**. Bacteria labeled with FITC and DAPI and attached to intestinal layer where intestinal villi were entirely observable were counted. Arrow shows an example of an enumerated bacterium. **(C)**: The position of each bacterium in picture **(A)** was identified (*x*_b_, *y*_b_) and the contour of the basal lumen of the mouse recto-anal junction to duodenum intestinal section was extracted by hand (∼300 points). The position of each bacterium along the intestine was obtained by identifying the point of the contour that was closest to the bacterium and oriented toward the center of the spiral. Micrograph obtained with AxioScan Z1 (Zeiss).

This gives the distance of each point of the organ from the recto-anal junction, *s* = 0, to the duodenum, *s* = L. The position of each bacterium along the organ was finally obtained by identifying the point of the contour that was the closest to the bacteria and oriented toward the center of the spiral. **Figure 1** is an example of a micrograph and the analysis performed on it.

### Statistical Analysis

For statistical analysis, experiments were conducted at least in five replicates. Mathematical regression for predicting EHEC localization in the milk phases were established using 3rd degree polynomial regressions.

A Wilcoxon signed-rank test was carried out to assess the effect of milk fat globules on adhesion of each EHEC strains to intestinal cell lines co cultured *in vitro*. Statistical significance was established at *p* < 0.01.

The significance of differences observed between EHEC strains excretion in mouse feces as a function of milk fat globule concentration was determined with the Wilcoxon signed rank test and was established at *p* < 0.05 or *p* < 0.001.

## Results

### Association of EHEC Strains With Milk Fat Phase

**FIGURE 2:**
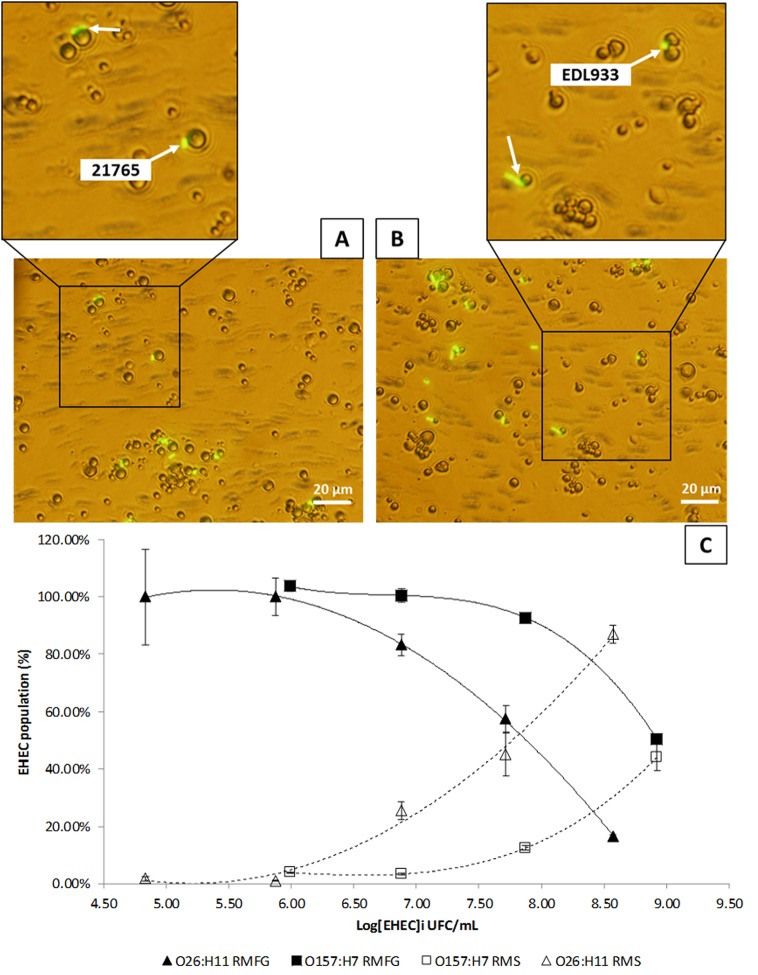
Enterohemorrhagic *Escherichia coli* (EHEC)–Milk Fat Globule (MFG) relationship in raw milk. **(A)** Epifluorescence micrograph of O26:H11 strain 21765 in raw milk. EHEC cells were labeled with anti-O26 antibodies coupled with FITC. **(B)** Epifluorescence micrograph of O157:H7 strain EDL933 in raw milk. EHEC cells were labeled with anti-O157 antibodies coupled with FITC. **(C)** Graph representation of the proportions of EHEC in raw milk fat globule (RMFG) phase and in raw milk skimmed (RMS) phase as a function of the initial EHEC concentration (CFU/mL) obtained by cream separation of raw milk fat globules containing 7.50 × 10^5^ to 8.40 × 10^8^ of EDL933 (CFU)/mL or 6.90 × 10^4^ to 3.70 × 10^8^ of 21765 (CFU)/mL. Key is shown in the figure.

To determine the localization of EHEC strains EDL933 and 21765 in raw milk, raw milk contaminated with 10 based serial dilutions of EHEC were creamed 24 h at 4°C to separate cream and skimmed phases. EHEC localization were then determined by plating each phase (Fat and skimmed phases) on specific agar medium. For the O26:H11 and O157:H7 strains, the proportion of the EHEC population in the milk fat phase decreased concomitantly with increasing initial EHEC concentration (**Figure 2C**). When the proportions of EHEC in the “fat” and “skimmed” phases were equal, that EHEC concentration was assigned as Concentration 50 (C_50_) of the MFG by EHEC strain. It took fewer EHEC in the fat phase to achieve this concentration with the O26:H11 strain than with O157:H7. The C_50_ of MFG was of 7.87 Log CFU/mL for EHEC O26:H11 strain 21765 and 8.98 Log CFU/ml for EHEC O157:H7 strain EDL933. The proposed mathematical model is a regression analysis on the data obtained by cream separation. Its fully explain the phenomenon observed in our experimental conditions (R^2^ comprised between 0.9937 and 1; **Table 2**).

**Table 2:** Mathematical regression models for distribution of EHEC O26:H11 strain 21765 and EHEC O157:H7 strain EDL933 in the different milk phases after cream separation.

EHEC	Milk phase	% of EHEC population in milk phase	*R*^2^
O157:H7	RMFG	= -0.0466x^3^+0.9444x^2^-6.3913x+15.4520	1.0000
	RMS	= 0.0173x^3^-0.3068x^2^+1.7916x-3.4001	1.0000
O26:H11	RMFG	= -0.0027x^3^-0.0294x^2^+0.5470x-0.6497	0.9999
	RMS	= -0.0007x^3^+0.0895x^2^-0.8815x+2.2560	0.9937


*All assays were performed in triplicate. RMFG, raw milk fat globule phase; RMS, raw milk skimmed phase*.

Cream separation controls performed in industrial skimmed milk showed that separation effect of EHEC in milk without fat globules was low (data not shown). The concentration of mesophilic facultative aerobic/anaerobic bacteria was between 3.64 × 10^4^ CFU/mL and 3.60 × 10^5^ CFU/mL and was below the saturation threshold of the MFGs, estimated at 10^8^ CFU/mL for both EHEC strains.

The micrograph of raw milk artificially contaminated with EHEC O26:H11 21765 and EHEC O157:H7 EDL933, labeled with antibodies coupled with fluorescein, shows that strains adhered to milk fat globules, regardless of the serotype (**Figures 2A,B**).

### Effect of MFGs on EHEC Adhesion to Intestinal Cells Lines Co-cultured *in Vitro*

**FIGURE 3:**
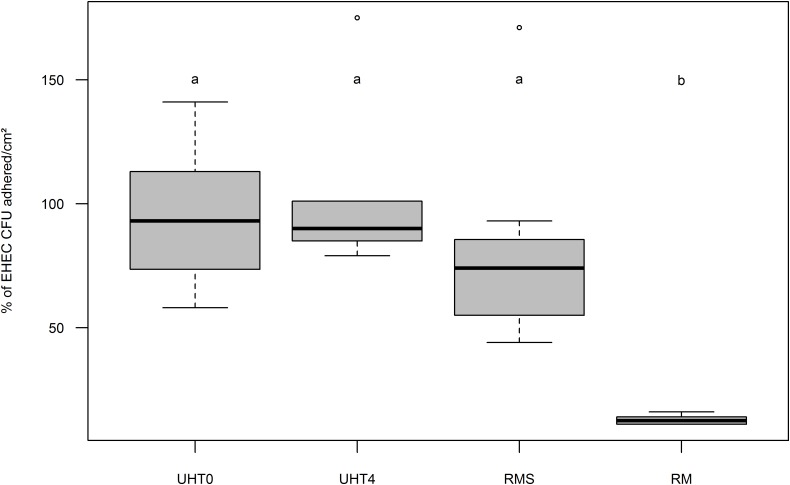
Boxplot of EHEC O157:H7 strain EDL933 adhesion percentage on Caco-2 and HT29-MTX enterocytes co-cultured *in vitro* as a function of fat globule concentration and milk treatment. Results are expressed as relative adherence calculated from the number of EHEC CFUs adhered to cell surfaces (cm^2^) +/– SEM of each condition compared to UHT0. UHT0, Ultra High Temperature treated skimmed milk (*n* = 12); UHT4, milk containing 4 g/kg of fat and treated by Ultra High Temperature (*n* = 6); RMS, Skimmed raw milk (*n* = 7); RM, Raw milk (*n* = 6). Groups (a and b) were defined for *p* < 0.001 according to Wilcoxon test. Circles represent aberrant data that were considered outliers.

**FIGURE 4:**
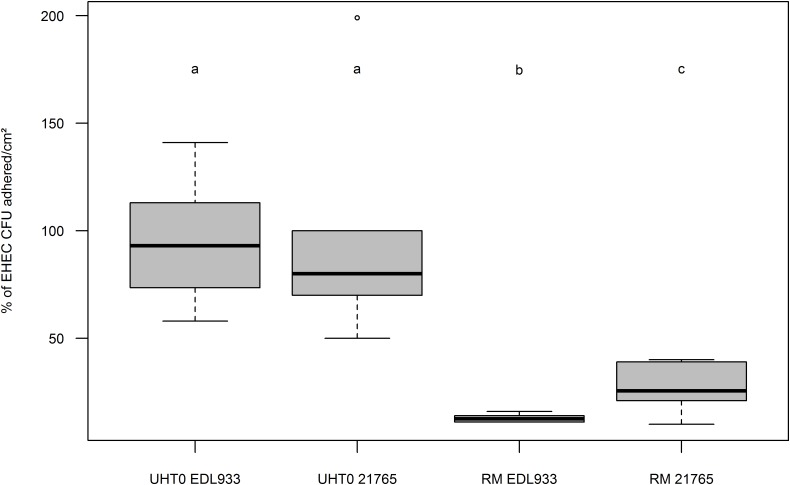
Boxplot of EHEC O26:H11 strain 21765 and EHEC O157:H7 strain EDL933 adhesion percentages to co-cultured Caco-2 and HT29-MTX enterocytes as function of fat globule concentration of the milk used. Results are expressed as EHEC CFUs adhered to cell surfaces (cm^2^) +/– SEM. UHT0, Ultra High Temperature treated skimmed milk (UHT0 EDL933 *n* = 12; UHT0 21765 *n* = 5); RM, Raw Milk (RM EDL933 *n* = 6; RM 21765 *n* = 6). Groups (a, b, and c) were defined for *p* < 0.001 according to Wilcoxon test. Circles represent aberrant data that were considered outliers.

To determine the influence of MFG and EHEC adherence *in vitro*, Caco-2/HT-29 co-cultures were incubated with artificially contaminated raw or UHT milk containing fat or not, and EHEC binding was evaluated by EHEC numeration after three consecutive washings. Adhesion of EHEC O157:H7 EDL933 to cells in the absence of MFGs (UHT skimmed milk and raw skimmed milk) was close to 100% for both milk treatments (**Figure 3**). Interestingly, the adhesion capability of EHEC cells to intestinal cell lines co-cultured *in vitro* in the presence of MFGs treated by UHT was similar to those without MFGs. Conversely, a significant reduction of EHEC O157:H7 EDL933 adhesion to co-cultured cells lines was observed in the presence of native MFGs (Raw milk). In the presence of natural MFGs, EHEC adhesion was significantly higher for EHEC O26:H11 21765 (28.28%) than for EHEC O157:H7 EDL933 (12.90%; **Figure 4**).

### Effect of Cheese MFGs on EHEC Excretion in Mouse Feces

An analytical image was produced using the “Swiss roll” histological section method and EHEC colonization data were obtained (a) from feces, (b) by intestinal quantification by rt-qPCR, and (c) by epifluorescence microscopy enumeration.

**FIGURE 5:**
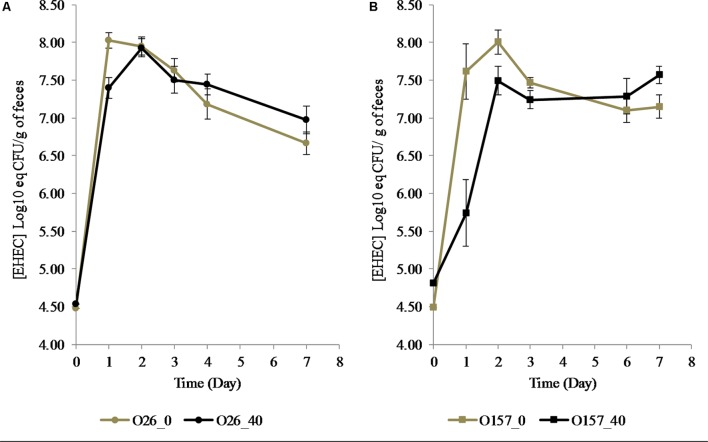
Excretion of EHEC O26:H11 strain 21765 str 225 (**A**: Circles) and O157:H7 EHEC strain EDL933 str 225 (**B**: Squares) in feces of mice fed with contaminated cheese with 40% (Black) or ∼0% (Gray) fat. Results obtained by RT-PCR are expressed as Log_10_ [EHEC] eq CFU/g of feces +/– SEM. ^∗∗^*p* < 0.01, Mann–Whitney test (*n* = 8 for each condition).

During cheese making, cheese microbiota density did not significantly differ between the cheeses over time (Supplementary Figure S3). Total bacteria in mouse feces were unchanged over time whatever the cheese they fed (**Table 3**). A significant decrease in EHEC O157:H7 EDL933 and EHEC O26:H11 21765 excretion in mouse feces was observed 1 day after consumption of EHEC-contaminated cheese, as a function of the MFG content of the cheese (**Figure 5**). Interestingly, with mice fed cheese containing 40% fat, this decrease in EHEC excretion was significantly greater for the O157:H7 strain than for the O26:H11 one. However, there was an increase in EHEC excretion 2 days post feeding regardless of strain and percentage of fat in the cheese fed (**Figure 5**). The maximum level of excretion was similar (**Figure 5**). Results of EHEC enumeration in mouse feces evaluated by rt-qPCR of O-antigen genes 7 days post-feeding were not significantly different for the two strains and the two types of cheese (**Table 4**).

**Table 3:** Time trend of total bacteria counts in feces of streptomycin-treated mice fed with cheese containing ∼0% (*n* = 8) or 40% (*n* = 8) of fat or not fed with cheese (*n* = 8).

Day	0	1	2	3	4	6	7
Microbiota_0%	8.55 +/- 0.21	9.19 +/- 0.26	8.88 +/- 0.42	9.26 +/- 0.27	8.95 +/- 0.33	9.21 +/- 0.12	9.03 +/- 0.46
Microbiota_40%	8.91 +/- 0.56	8.73 +/- 0.60	9.01 +/- 0.43	9.10 +/- 0.26	9.12 +/- 0.27	9.08 +/- 0.33	8.96 +/- 0.81
Microbiota_NC	9.00 +/- 0.33	9.12 +/- 0.35	8.95 +/- 0.56	9.41 +/- 0.24	8.86 +/- 0.62	9.12 +/- 0.19	9.06 +/- 0.50


**Results were obtained by rt-PCR on rRNA 16S coding gene as described in Section “Materials and Methods.” They are expressed as mean Log_10_ [bacteria] eqCFU/g of feces +/-SEM. Microbiota_0%, microbiota from mice fed with ∼0% fat cheese; Microbiota_40%, microbiota from mice fed with 40% fat cheese. Microbiota_NC, microbiota from mice not fed with cheese*.*

**Table 4:** Concentration of EHEC O26:H11 strain 21765 SR225 (O26) or EHEC O157:H7 strain EDL933 SR225 (O157) in feces (*n* = 8 for each condition) and in recto-anal junction to duodenum section of mouse intestine (*n* = 5 for each condition) were estimated by rt-qPCR (Log_10_ [EHEC] eqCFU/g+/-SEM), 7 days after feeding with contaminated cheese containing fat (40%) or not (∼0%).

	Log_10_[EHEC] eqCFU/g of feces (rt-qPCR)	Log_10_[EHEC] eqCFU/g of organ (rt-qPCR)	Log_10_ EHEC (CFU) adhered to mouse recto-anal-duodenum section (Microscopy)	Length of recto-anal junction-duodenum section (cm)
O26_0	6.66 +/- 0.15	6.27 +/- 0.24	3.01	29.79
O26_40	6.97 +/- 0.18	6.20 +/- 0.17	3.06	28.50
O157_0	7.15 +/- 0.15	5.83 +/- 0.33	3.02	28.36
O157_40	7.57 +/- 0.12	6.26 +/- 0.23	3.19	30.37
Average	7.09	6.14	3.07	–


**They were also counted in mouse intestine by epifluorescence microscopy analysis (see section “Materials and Methods”). The lengths (cm) of the recto-anal junction to duodenum sections are also shown*.*

### Implantation of EHEC Strains in Mouse Intestinal Tract 7 Days Post Inoculation

To compare results obtained by both methods microscopy and rt-qPCR, the EHEC count obtained on the 4 μm-thick sections of mouse recto-anal junction to duodenum by microscopy was calculated in order to be representative of the whole intestinal tract. The total area of small and large intestine from 4-weeks old CD1 mice was estimated as stated by Wołczuk et al. (2011) then total EHEC/organ was calculated. EHEC enumeration in whole intestine of mice obtained by microscopy and rt-qPCR were compared and showed that results were comparable with each other (**Table 5**).

**Table 5:** Enterohemorrhagic *Escherichia coli* EHEC enumeration in whole mice intestine by microscopy and rt-qPCR.

	EHEC/organ^∗^ counted by microscopy			EHEC/organ counted by rt-qPCR	
		
	Average^∗∗^	Minimum^∗∗^	Maximum^∗∗^	Average (*n* = 5) +/- SEM	Organ mass (g)
O26_0	7.01	6.8	7.15	6.59 +/- 0.24	2.11
O26_40	7.08	6.87	7.22	6.40 +/- 0.17	1.59
O157_0	7.04	6.83	7.18	6.19 +/- 0.33	2.27
O157_40	7.18	6.97	7.32	6.55 +/- 0.23	1.95


**^∗^Organ area was calculated considering 4-weeks old CD1 mice morphometric parameters described by Wołczuk et al. (2011). ^∗∗^Average, minimum and maximum of EHEC/organ counted by microscopy were calculated considering parameters described by Wołczuk et al. (2011)*.*

**FIGURE 6:**
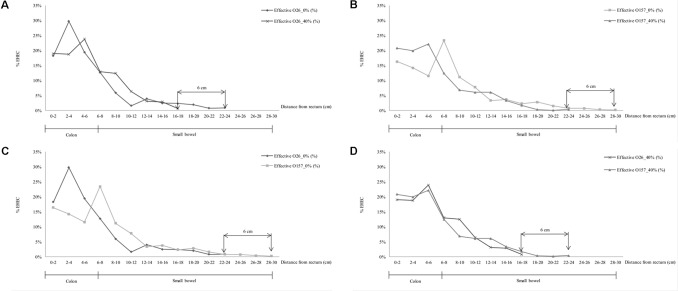
Localization and density (%) of EHEC O157:H7 EDL933 SR225 (O157) and O26:H11 21765 SR225 (O26) in murine intestinal tract from recto-anal junction to upper intestine (cm) as a function of the presence (40%) or limited amount (∼0%) of MFGs in contaminated cheese, 7 days after inoculation. **(A)** Localization of O26:H11 in mouse intestine according to MFGs in the contaminated cheese fed (40 or ∼0%) **(B)** O157:H7 strain localization in mouse intestine according to MFGs in the contaminated cheese fed. **(C)** Localization of O26:H11 and O157:H7 in mouse intestine after feeding with contaminated ∼0% fat cheese; **(D)** O26:H11 and O157:H7 strains localization in mouse intestinal tract after feeding with contaminated 40% fat cheese.

As described above, the rate of EHEC excretion in mouse feces 7 days post-feeding, did not depend on cheese fat content. This excretion rate was approximately 7.09 Log CFU/g of feces (**Table 4**). Similarly, examination of the recto-anal junction to duodenum section of mouse intestine 7 days post inoculation showed no difference in EHEC adhesion between assays (∼6.14 Log CFU/g of organ on five mice; **Table 4**). Whole mouse intestines were scanned by epifluorescence microscopy in order to estimate the localization of EHEC implantation in the intestinal tracts of mice as a function of the fat concentration in the cheese used for feeding (**Figures 1**, **6**). No differences in enumeration of adhered EHEC were observed from the analysis of whole-intestine “Swiss rolls” (∼3.07 Log CFU; **Table 4**).

The localization of EHEC concentrations in the intestines of mice differed according to strain and to cheese fat content. The distance of primo-implantation of the EHEC O26:H11 strain was comprised between 6 and 8 cm more distal from the recto-anal junction in the absence of MFGs from the cheese fed to the mice than in their presence (**Figure 6A**; 22–24 cm for ∼0% vs. 16–18 cm for 40%). Similarly, data for the O157:H7 strain in the absence of MFGs showed a primo implantation between 6 and 8 cm earlier than in their presence (**Figure 6B**; 28–30 cm for ∼0% vs. 22–24 cm for 40%). Maximum densities of EHEC O157:H7 in the presence or absence of MFGs in cheese consumed were similar (**Figure 6B**; 23% for ∼0 and 22% for 40%) but differed with EHEC O26:H11 (**Figure 6A**; 24% for ∼0 and 30% for 40%). Differences in implantation were observed between the two strains (**Figure 6C**). Where amounts of MFG in cheese the diet were small (∼5%), the primo implantation of EHEC O157:H7 was 6 cm more distal from murin recto-anal junction than that of EHEC O26:H11 21765. Similarly, the maximum density was obtained at a distance of 2 to 4 cm from the rectum for EHEC O26:H11 21765 but between 6 and 8 cm for the O157:H7 EDL933. Interestingly, while the counts of EHEC cells were similar for the two strains (**Table 4**), the maximum density was 30% for EHEC O26:H11 21765 vs. 23% for the O157:H7 strain (**Figure 6C**). In presence of 40% MFGs in the cheese consumed, the implantation profiles of the two EHEC were similar (**Figure 6D**). Nevertheless, the primo implantation of EHEC O157:H7 was 6 cm more distal from the recto-anal junction than that of the O26:H11 strain.

### EHEC Association With MFGM as a Natural EHEC Receptor Analog

**FIGURE 7:**
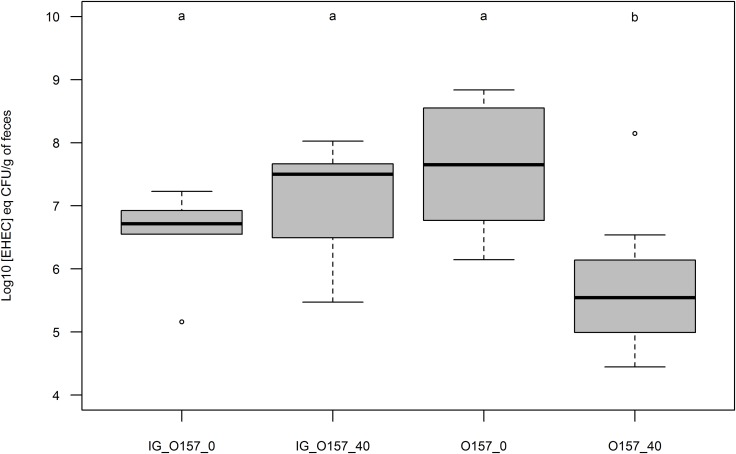
Boxplot representation of EHEC concentration in mouse feces 24 h post inoculation (Log_10_ [EHEC] eqCFU/g of feces). Mice were pre-fed with uncontaminated cheeses containing fat (40) or not (∼0) during 7 days. Mice inoculation of O157:H7 strain EDL933 SR225 was done intra-gastrically (IG; *n* = 5 for each condition) or by feeding contaminated cheese containing fat (40; *n* = 8) or not (∼0; *n* = 8). Groups were assigned as *p* < 0.01 by Mann Whitney test.

Because adhesion was more strongly reduced with EHEC O157:H7, we used this strain to perform an adhesion assay by intra-gastric inoculation of a suspension after a 1-week diet of cheese containing ∼0 or 40% fat. Mice pre-fed for 7 days with cheese containing fat or not and intra-gastrically inoculated with an EHEC suspension displayed similar rates of EHEC excretion 1-day post inoculation (**Figure 7**). EHEC excretion was not significantly different for mice pre-fed with ∼0% fat cheese and fed with EHEC contaminated cheese ∼0% MFG, but was significantly lower for mice pre-fed with 40% fat cheese and fed with contaminated 40% fat cheese. **Figure 7** shows that pre-feeding animals with a high-fat cheese before intra-gastric EHEC inoculation did not in itself protect mice from EHEC intestinal colonization.

## Discussion

Microscopy observations demonstrated that EHEC cells displayed a tropism for native MFGs. Our findings are in agreement with those reported for several other entero-pathogenic bacteria that are associated with MFGM (Lopez et al., 2006; Ly-Chatain et al., 2010; Douellou et al., 2017). Our results show that EHEC were clearly associated with the fat phase of raw milk. MFGM was saturated with a lesser amount of EHEC O26:H11 than of EHEC O157:H7. This may be because EHEC O157:H7 EDL933 has more adhesion factors than EHEC O26:H11 strain 21765, based on genome analysis (Perna et al., 1998; Galia et al., 2015). Moreover, O157:H7 EHEC strains appears to express more adhesion factors than O26:H11 strains (Low et al., 2006; Bardiau et al., 2009).Thus, for a given EHEC concentration, the presence of more adhesion appendices at the surface of EHEC O157:H7 strain EDL933 might allow it to attach to MFGM more easily than the O26:H11 strain 21765. Glycoconjugates on the fat-aqueous interface of the MFGs presents mimetic analogs of cell receptors involved in recognition of EHEC strains (Ross et al., 2016; Douellou et al., 2017). Nevertheless, there were always EHEC cells in the raw milk skimmed phase, whatever the initial EHEC concentration. The mathematical model obtained in this work is a regression of dataset obtained by the milk-cream separation assays. These regressions showed that for an EHEC concentration less than 6 Log_10_ CFU/mL, almost 99.9% of the bacteria are located in the fat phase. The natural EHEC contamination rate in milk is currently less than 0.5 Log CFU/mL (Perrin et al., 2015). This may suggest that EHEC in naturally contaminated milk are mainly located at the fat-aqueous phase interface. The knowledge this work provides may be useful for milk risk-assessment managers, who could focus on the milk fat phase when analyzing milk to detect potentially highly pathogenic STEC.

Our study report that native MFGs may limit the adhesion of two EHEC strains from serotypes O157:H7 and O26:H11 to Caco-2/HT29-MTX (9:1) intestinal cells in a co-cultured monolayer. EHEC adhesion to intestinal cells cultured *in vitro* suggests that the integrity of the MFGM-linked glycoconjugates is a necessary condition for inhibiting EHEC adhesion to cells (Brewster and Paul, 2016; Ross et al., 2016). The anti-adhesive properties of MFGs depend on fat structure and integrity. Milk treatment has an impact on the integrity of the MFGM and its associated glycoconjugates (Lopez et al., 2011; Douellou et al., 2017). Pasteurization and homogenization keep fat dispersed in the aqueous phase but also partially disrupt MFGM and the glycoconjugates on its surface (Lopez et al., 2006; Douellou et al., 2017). Interestingly, our EHEC O157:H7 strain’s adhesion was inhibited more strongly in the presence of native MFGM than was that of EHEC O26:H11. These observations are concordant with data obtained from cream separation assays that also demonstrated different tropism from the O157:H7 strain to MFG surface compared to the O26:H11 one. Thus, as more EDL933 O157:H7 bacteria were adhered to MFGM, inhibition of this strain adhesion to intestinal cells monolayer was stronger. Moreover, weak interactions (e.g., Van der Walls interaction, ionic bonds) might be involved in EHEC adhesion to MFGs, as already suggested for *Lactobacillus reuteri* (Brisson et al., 2010). These weak interactions are directly correlated with the cell charge of the bacteria (Brisson et al., 2010) and to the ionic condition, that determine the strength of the interactions. The presence of extracellular structures such as fimbriae and/or lipopolysaccharides may influence the hydrophobicity and cell charge of EHEC strains (Boyer et al., 2007), and these may be important factors in bacterial attachment to MFGM surfaces. Once again, these kinds of weak bonds may be more involved in the phenomenon with the EHEC O157:H7 strain, since it presents more adhesion factors than EHEC belonging to O26:H11 serotype (Low et al., 2006; Ogura et al., 2007; Bardiau et al., 2009).

*Escherichia coli* can be used as an indicator of hygiene in dairy products industry. Several works demonstrated that *E. coli* and STEC multiplication and survival must be dependent of the physicochemical characteristic of the dairy matrix during cheese making (Peng et al., 2013; Miszczycha et al., 2014; Kim et al., 2018). Indeed, several cheeses technological process limited (e.g., cooked cheese, blue cheese, lactic cheese) or improved (uncooked pressed cheese, mold cheese) STEC survival and multiplicity. In our work, we chose the “most risky scenario” by studying effect of EHEC adhesion after consumption of contaminated unpasteurized uncooked pressed cheese in order to increase the probability of EHEC survival and thus increase potential mouse intestinal tract colonization by the pathogen.

In our study, EHEC O157:H7 and EHEC O26:H11 excretion occurred 2 days post-feeding in mice fed with 40% fat cheese and 1 day post-feeding in mice fed with ∼0% fat cheese. This inhibition of excretion was significantly greater for the O157:H7 strain. These data confirm our results from milk fat cream separation and culture cell adhesion assays. The level of EHEC O26:H11 in mouse feces 1 day post-feeding in the presence of MFGs in the cheese matrix was equivalent to that of EHEC O157:H7 in mice fed cheese without MFGs. The presence of MFGs in the cheese may limit EHEC adhesion capabilities to differing degrees. The MFG effect appears to be stronger for the O157:H7 strain. This may be due to the fact that O26:H11 displays better fitness than O157:H7 in the digestive environment (Miszczycha et al., 2014) and the fact that O157:H7 possesses stronger adhesion factor expression capabilities than O26:H11 (Ogura et al., 2007). It has been shown that, 1 day post inoculation, symptoms caused by attenuated diarrheagenic *E. coli* were less severe in patients pre-fed an MFGM-rich diet and there was complete recovery after 2 days (Ten Bruggencate et al., 2016). Our study complements these observations with the hypothesis that the presence of MFGM components in the patient’s gut may inhibit pathogenic *E. coli* adhesion to target intestinal cells, allowing them to be eliminated in the first 24 h. This report fills the gap of knowledge to assess the anti-adhesion effects of MFGs against the two strains of EHEC O157:H7 and O26:H11 colonization in mice after feeding them with contaminated cheese.

Moreover, our results were obtained in sub-physiological conditions since contaminations of raw milk by EHEC *in situ* rarely occurs at more than 0.5 CFU/mL before cheese making (Perrin et al., 2015). Our results suggest that for lesser amount of EHEC per grams of cheese, presence of MFGM might protect consumer by increasing the infective dose of EHEC.

Pre-feeding streptomycin-treated mice with cheese containing MFGs does not seem to protect them against EHEC O157:H7 colonization when intra-gastrically inoculated with a suspension. However, those inoculated by feeding them EHEC-contaminated high-fat cheese excrete fewer EHEC 1 day post inoculation. This suggests that native MFGs possess EHEC receptor-like features and do not compete with EHEC for *in situ* adhesion site. Nevertheless, the absence of differences between treatments 2 days post-feeding suggests that the inhibition of EHEC adhesion by MFGs may not be sufficient to protect the host against EHEC colonization of the intestine. Interestingly, 7 days post infection, no difference was observed in numbers of EHEC cells adhering to mouse intestinal tracts. Mice intestinal transit time follows an exponential path and is about 307.5 +/- 15 min (Kashyap et al., 2013). This suggests that MFGs are digested and/or evacuated from the mouse intestinal tract a few hours post-feeding. Thus, even if fewer EHEC were initially implanted in the presence of MFGs, their growth in the intestine could make up the initial shortfall over time.

In the absence of MFGs, EHEC O157:H7 tends to be mainly located in the first part of the colon while EHEC O26:H11 seemed to be located in the middle/end parts. The main adhesion sites were not the same for the two EHEC strains. Interestingly, the presence of MFGs in the contaminated cheese caused the site of primo-implantation to occur 6 cm further along the gut, for both EHEC strains; it appeared to modify the adhesion sites of these EHEC strains in the colon. Moreover, the O157:H7 strain adhered more distally from the recto-anal junction than the O26:H11 strain. It can be hypothesized that EHEC O157:H7 expressed more adhesion factors than EHEC O26:H11 (Low et al., 2006; Ogura et al., 2007; Bardiau et al., 2009) in these conditions, but no comparable data are available in the literature. Whatever the type of cheese used, EHEC O157:H7 colonized a larger intestinal area than EHEC O26:H11. The fact that EHEC cells colonize the upper intestine due to particular environment that enhances their development and survival allowed them to grow and release newly formed bacteria. These released bacteria in the lumen of the intestine could recognize new site of colonization downstream of the initial recognized site in the intestinal tract. This could give EHEC O157:H7 more chance to find its intestinal adhesion receptors (such as Peyer’s patches) and so cause illness. *Ad contrario*, feeding mice with 40% fat cheese reduces the intestinal surface contaminated with O157:H7 might thus decrease the severity of illness.

Our results from whole-intestine scanning show that the predominant EHEC colonization sites were in the terminal ileal loop and large intestine, as has been reported elsewhere (Nataro and Kaper, 1998; Etienne-Mesmin et al., 2011). MUC2 is the main mucin in this part of the gut but relative adhesion would be affected by the different mucins. Mucins on the MFGM surface are mainly MUC1 and MUC15. The fact that EHEC showed tropism for the MUC glycoproteins family (Ye et al., 2015) suggests that the presence of MFGM-MUC1 might act as efficient decoy for EHEC intestinal adhesion.

Most of the epidemic cases due to EHEC and linked to dairy products were related to raw milk products consumption (Guzman-Hernandez et al., 2016; Nobili et al., 2016) and few have been linked to pasteurized dairy products (Upton and Coia, 1994). These last are generally linked to contact surfaces contamination with EHEC strain(s) from food located after the pasteurizer (Boor et al., 2017). Pasteurization kills bacteria, improving safety product, and modifies the structure of MFGM (Lopez, 2011). It has been shown that the absence of endogen microbiota improve EHEC multiplication and might thus increase the exposure of consumers to this pathogen (Galia et al., 2017). In another hand, pasteurization changes the MFGM structure which could be involved in the inhibition of EHEC adhesion to intestinal tract as demonstrated here. Thus, it can be hypothesized that while a post-pasteurization contamination with bacterial pathogens remains an issue, even in the modern dairy industry, they might also be more risky since these products would not still be able to protect consumers against pathogens as raw milk dairy products do.

To conclude, MFG in cheese in the diet could be involved in the reduction of EHEC severity. Future research must be done on a greater number of strains to strengthen the difference between the EHEC serotypes. In addition, experimental evidence of the difference in the number of adhesion factors should be obtained to confirm the proposed hypothesis. While the hypothesis of MFGM carbohydrates might be involved in the inhibition of EHEC adhesion to the intestinal tract, no evidence has been offered in this work. Future investigation might focus on identification of molecules involved in the inhibition of EHEC adhesion to the intestinal tract. Blocking or inhibition of adhesion appendages by suitable MFGM-derived molecules open the way for new strategies to prevent or treat microbial diseases by anti-adhesion therapy.

## Author Contributions

Each author has contributed to the paper based on the following tasks: TD, WG, M-CM, and DS-T: conception or design of the work. TD, SK, MB, TM, NM, and SB: data collection. TD, WG, RB, M-CM, and DS-T: data analysis and interpretation. TD, RB, M-CM, DS-T: drafting the article. WG, SB, SK, TM, and NM: critical revision of the article. All authors approved the final version of the manuscript to be published.

## Conflict of Interest Statement

The authors declare that the research was conducted in the absence of any commercial or financial relationships that could be construed as a potential conflict of interest.

## Footnotes

1 http://www.ebi.ac.uk/ena/data/view

## References

[B1] Bao Y. Zhu L. Newburg D. S. (2007). Simultaneous quantification of sialyloligosaccharides from human milk by capillary electrophoresis. 370 206–214. 10.1016/j.biotechadv.2011.08.021.Secreted 17761135 PMC2441650

[B2] Bardiau M. Labrozzo S. Mainil J. G. (2009). Putative adhesins of enteropathogenic and enterohemorrhagic *Escherichia coli* of serogroup O26 isolated from humans and cattle. 47 2090–2096. 10.1128/JCM.02048-08 19403767 PMC2708474

[B3] Boor K. J. Wiedmann M. Murphy S. Alcaine S. (2017). A 100-year review: microbiology and safety of milk handling. 100 9933–9951. 10.3168/jds.2017-12969 29153181

[B4] Boyer R. R. Sumner S. S. Williams R. C. Pierson M. D. Popham D. L. Kniel K. E. (2007). Influence of curli expression by *Escherichia coli* O157:H7 on the cell’s overall hydrophobicity, charge, and ability to attach to lettuce. 70 1339–1345. 10.4315/0362-028X-70.6.1339 17612061

[B5] Brewster J. D. Paul M. (2016). Short communication: improved method for centrifugal recovery of bacteria from raw milk applied to sensitive real-time quantitative PCR detection of *Salmonella* spp. 99 3375–3379. 10.3168/jds.2015-9655 26971150

[B6] Brisson G. Payken H. F. Sharpe J. P. Jiménez-Flores R. (2010). Characterization of *Lactobacillus reuteri* interaction with milk fat globule membrane components in dairy products. 58 5612–5619. 10.1021/jf904381s 20377223

[B7] Buchholz U. Bernard H. Werber D. Böhmer M. M. Remschmidt C. Wilking H. (2011). German outbreak of *Escherichia coli* O104:H4 associated with sprouts. 365 1763–1770. 10.1056/NEJMoa1106482 22029753

[B8] Chen X. M. Elisia I. Kitts D. D. (2010). Defining conditions for the co-culture of Caco-2 and HT29-MTX cells using Taguchi design. 61 334–342. 10.1016/j.vascn.2010.02.004 20159047

[B9] Croxen M. A. Finlay B. B. (2010). Molecular mechanisms of *Escherichia coli* pathogenicity. 8 26–38. 10.1038/nrmicro2265 19966814

[B10] Douellou T. Montel M. C. Sergentet D. T. (2017). Anti-adhesive properties of bovine oligosaccharides and bovine milk fat globule membrane-associated glycoconjugates against bacterial food enteropathogens. 100 3348–3359. 10.3168/jds.2016-11611 28161162

[B11] EFSA and ECDC. (2015). The European Union summary report on trends and sources of zoonoses, zoonotic agents and food-borne outbreaks in 2014. 13:4329 10.2903/j.efsa.2015.4329 PMC7009540 32625785

[B12] EFSA Panel on Biological Hazards (2015). Scientific opinion on the public health risks related to the consumption of raw drinking milk. 13 3940–4035. 10.2903/j.efsa.2015.3940

[B13] Etienne-Mesmin L. Chassaing B. Sauvanet P. Denizot J. Blanquet-Diot S. Darfeuille-Michaud A. (2011). Interactions with M cells and macrophages as key steps in the pathogenesis of enterohemorragic *Escherichia coli* infections. 6:e23594. 10.1371/journal.pone.0023594 21858177 PMC3157389

[B14] Galia W. Leriche F. Cruveiller S. Garnier C. Navratil V. Dubost A. (2017). Strand-specific transcriptomes of enterohemorrhagic *Escherichia coli* in response to interactions with ground beef microbiota: interactions between microorganisms in raw meat. 18:574. 10.1186/s12864-017-3957-2 28774270 PMC5543532

[B15] Galia W. Mariani-Kurkdjian P. Bastien S. Loukiadis E. Blanquet-Diot S. Leriche F. (2015). Genome sequence and annotation of a human infection isolate of *Escherichia coli* O26:H11 involved in a raw milk cheese outbreak. 3:e01568–14 10.1128/genomeA.01568-14 PMC4335332 25700408

[B16] Guri A. Griffiths M. Khursigara C. M. Corredig M. (2012). The effect of milk fat globules on adherence and internalization of *Salmonella* Enteritidis to HT-29 cells. 95 6937–6945. 10.3168/jds.2012-5734 23021758

[B17] Guzman-Hernandez R. Contreras-Rodriguez A. Hernandez-Velez R. Perez-Martinez I. Lopez-Merino A. Zaidi M. B. (2016). Mexican unpasteurised fresh cheeses are contaminated with *Salmonella* spp., non-O157 Shiga toxin producing *Escherichia* coli and potential uropathogenic *E. coli* strains: A public health risk. 237 10–16. 10.1016/J.IJFOODMICRO.2016.08.018 27541977

[B18] Hentges D. Que J. Casey S. Stein A. (1984). The influence of streptomycin on colonization resistance in mice. 14 53–62.

[B19] Hilgendorf C. Spahn-Langguth H. Regårdh C. G. Lipka E. Amidon G. L. Langguth P. (2000). Caco-2 versus Caco-2/HT29-MTX co-cultured cell lines: permeabilities via diffusion, inside- and outside-directed carrier-mediated transport. 89 63–75. 10.1002/(SICI)1520-6017(200001)89:1<63::AID-JPS7<3.0.CO;2-6 10664539

[B20] Kaper J. B. Nataro J. P. Mobley H. L. T. (2004). Pathogenic *Escherichia coli*. 2 123–140. 10.1038/nrmicro818 15040260

[B21] Kashyap P. C. Marcobal A. Ursell L. K. Larauche M. Duboc H. Earle K. A. (2013). Complex interactions among diet, gastrointestinal transit, and gut microbiota in humanized mice. 144 967–977. 10.1053/j.gastro.2013.01.047 23380084 PMC3890323

[B22] Kim N. H. Lee N. Y. Kim M. G. Kim H. W. Cho T. J. Joo I. S. (2018). Microbiological criteria and ecology of commercially available processed cheeses according to the product specification and physicochemical characteristics. 106 468–474. 10.1016/J.FOODRES.2018.01.014 29579949

[B23] Klemm P. Vejborg R. M. Hancock V. (2010). Prevention of bacterial adhesion. 88 451–459. 10.1007/s00253-010-2805-y 20694794

[B24] Leatham M. P. Banerjee S. Autieri S. M. Mercado-Lubo R. Conway T. Cohen P. S. (2009). Precolonized human commensal *Escherichia coli* strains serve as a barrier to *E. coli* O157:H7 growth in the streptomycin-treated mouse intestine. 77 2876–2886. 10.1128/IAI.00059-09 19364832 PMC2708557

[B25] Lesuffleur T. Barbat A. Dussaulx E. Zweibaum A. (1990). Growth adaptation to methotrexate of HT-29 human colon carcinoma cells is associated with their ability to differentiate into columnar absorptive and mucus-secreting cells. 50 6334–6343. 2205381

[B26] Lesuffleur T. Porchet N. Aubert J. P. Swallow D. Gum J. R. Kim Y. S. (1993). Differential expression of the human mucin genes MUC1 to MUC5 in relation to growth and differentiation of different mucus-secreting HT-29 cell subpopulations. 106(Pt 3), 771–783. 8308060 10.1242/jcs.106.3.771

[B27] Lopez C. (2011). Milk fat globules enveloped by their biological membrane: unique colloidal assemblies with a specific composition and structure. 16 391–404. 10.1016/j.cocis.2011.05.007

[B28] Lopez C. Briard-Bion V. Ménard O. Beaucher E. Rousseau F. Fauquant J. (2011). Fat globules selected from whole milk according to their size: different compositions and structure of the biomembrane, revealing sphingomyelin-rich domains. 125 355–368. 10.1016/j.foodchem.2010.09.005

[B29] Lopez C. Maillard M. B. Briard-Bion V. Camier B. Hannon J. A. (2006). Lipolysis during ripening of emmental cheese considering organization of fat and preferential localization of bacteria. 54 5855–5867. 10.1021/jf060214l 16881687

[B30] Loukiadis E. Callon H. Mazuy-Cruchaudet C. Vallet V. Bidaud C. Ferré F. (2012). Surveillance des *Escherichia coli* producteurs de shigatoxines (STEC) dans les denrées alimentaires en France (2005-2011). 55 2–9.

[B31] Low A. S. Holden N. Rosser T. Roe A. J. Constantinidou C. Hobman J. L. (2006). Analysis of fimbrial gene clusters and their expression in enterohaemorrhagic *Escherichia coli* O157:H7. 8 1033–1047. 10.1111/j.1462-2920.2006.00995.x 16689724

[B32] Luo S. McNeill M. Myers T. G. Hohman R. J. Levine R. L. (2008). Lon protease promotes survival of *Escherichia coli* during anaerobic glucose starvation. 189 181–185. 10.1007/s00203-007-0304-z 17891379 PMC3397802

[B33] Ly-Chatain M. H. Le M. L. Thanh M. L. Belin J. M. Waché Y. (2010). Cell surface properties affect colonisation of raw milk by lactic acid bacteria at the microstructure level. 43 1594–1602. 10.1016/j.foodres.2010.04.019

[B34] Miszczycha S. D. Perrin F. Ganet S. Jamet E. Tenenhaus-Aziza F. Montel M. C. (2013). Behavior of different Shiga toxin-producing *Escherichia coli* serotypes in various experimentally contaminated raw-milk cheeses. 79 150–158. 10.1128/AEM.02192-12 23087038 PMC3536096

[B35] Miszczycha S. D. Thévenot J. Denis S. Callon C. Livrelli V. Alric M. (2014). Survival of *Escherichia coli* O26:H11 exceeds that of *Escherichia coli* O157:H7 as assessed by simulated human digestion of contaminated raw milk cheeses. 172 40–48. 10.1016/j.ijfoodmicro.2013.11.029 24361831

[B36] Moolenbeek C. Ruitenberg E. J. (1981). The “Swiss roll”: a simple technique for histological studies of the rodent intestine. 15 57–59. 10.1258/002367781780958577 7022018

[B37] Nataro J. P. Kaper J. B. (1998). Diarrheagenic *Escherichia coli*. 11 142–201. 10.1128/cmr.11.1.142 PMC121379 9457432

[B38] Nobili G. Franconieri I. Basanisi M. G. La Bella G. Tozzoli R. Caprioli A. (2016). Short communication: isolation of shiga toxin-producing *Escherichia coli* in raw milk and mozzarella cheese in southern Italy. 99 7877–7880. 10.3168/jds.2016-11613 27522413

[B39] Ofek I. Hasty D. L. Sharon N. (2003). Anti-adhesion therapy of bacterial diseases: prospects and problems. 38 181–191. 10.1016/S0928-8244(03)00228-1 14522453

[B40] Ogura Y. Ooka T. Asadulghani M. Terajima J. Nougayrède J.-P. Kurokawa K. (2007). Extensive genomic diversity and selective conservation of virulence-determinants in enterohemorrhagic *Escherichia coli* strains of O157 and non-O157 serotypes. 8:R138. 10.1186/gb-2007-8-7-r138 17711596 PMC2323221

[B41] Peng S. Hoffmann W. Bockelmann W. Hummerjohann J. Stephan R. Hammer P. (2013). Fate of Shiga toxin-producing and generic *Escherichia coli* during production and ripening of semihard raw milk cheese. 96 815–823. 10.3168/jds.2012-5865 23245958

[B42] Park J. W. Crowley D. E. (2006). Dynamic changes in *nahAc* gene copy numbers during degradation of naphthalene in PAH-contaminated soils. 72 1322–1329. 10.1007/s00253-006-0423-5 16804694

[B43] Perelle S. Dilasser F. Grout J. Fach P. (2004). Detection by 5′-nuclease PCR of Shiga-toxin producing *Escherichia coli* O26, O55, O91, O103, O111, O113, O145 and O157:H7, associated with the world’s most frequent clinical cases. 18 185–192. 10.1016/j.mcp.2003.12.004 15135453

[B44] Perna N. T. Mayhew G. F. Posfai G. Elliott S. Donnenberg M. S. Kaper J. B. (1998). Molecular evolution of a pathogenicity island from enterohemorrhagic *Escherichia coli* O157:H7. 66 3810–3817. 9673266 10.1128/iai.66.8.3810-3817.1998 PMC108423

[B45] Perrin F. Tenenhaus-Aziza F. Michel V. Miszczycha S. Bel N. Sanaa M. (2015). Quantitative risk assessment of haemolytic and uremic syndrome linked to O157: H7 and non-O157: H7 Shiga-toxin producing *Escherichia coli* strains in raw milk soft cheeses. 35 109–128. 10.1111/risa.12267 25156259

[B46] Ross S. A. Lane J. A. Kilcoyne M. Joshi L. Hickey R. M. (2016). Defatted bovine milk fat globule membrane inhibits association of enterohaemorrhagic *Escherichia coli* O157:H7 with human HT-29 cells. 59 36–43. 10.1016/j.idairyj.2016.03.001

[B47] Sprong R. C. Hulstein M. F. E. Van Der Meer R. (2002). Bovine milk fat components inhibit food-borne pathogens. 12 209–215. 10.1016/S0958-6946(01)00139-X

[B48] Struijs K. Van De Wiele T. Le T. T. Debyser G. Dewettinck K. Devreese B. (2013). Milk fat globule membrane glycoproteins prevent adhesion of the colonic microbiota and result in increased bacterial butyrate production. 32 99–109. 10.1016/j.idairyj.2013.05.001

[B49] Ten Bruggencate S. J. Frederiksen P. D. Pedersen S. M. Floris-Vollenbroek E. G. Lucas-van de Bos E. van Hoffen E. (2016). Dietary milk-fat-globule membrane affects resistance to diarrheagenic *Escherichia coli* in healthy adults in a randomized, placeboa-controlled, double-blind study. 146 249–255. 10.3945/jn.115.214098 26701793

[B50] Upton P. Coia J. E. (1994). Outbreak of *Escherichia coli* O157 infection associated with pasteurised milk supply. 344:1015. 10.1016/S0140-6736(94)91670-5 7934390

[B51] Wang X. Hirmo S. Willen R. Wadstrom T. (2001). Inhibition of *Helicobacter pylori* infection by bovine milk glycoconjugates in a BAlb/cA mouse model. 50 430–435. 10.1099/0022-1317-50-5-430 11339250

[B52] Wołczuk K. Wilczyńska B. Jaroszewska M. Kobak J. (2011). Morphometric characteristics of the small and large intestines of Mus musculus during postnatal development. 70 252–259. 22117242

[B53] Ye J. Song L. Liu Y. Pan Q. Zhong X. Li S. (2015). Core 2 mucin-type O-glycan is related to EPEC and EHEC O157:H7 adherence to human colon carcinoma HT-29 epithelial cells. 60 1977–1990. 10.1007/s10620-015-3548-5 25701318

