# Supplementary material for: Milk Fat Globules Hamper Adhesion of Enterohemorrhagic Escherichia coli to Enterocytes: In Vitro and in Vivo Evidence
Source: Front Microbiol. 2018 May 15;9:947. doi: 10.3389/fmicb.2018.00947 (PMC5963252; doi:10.3389/fmicb.2018.00947)
Supplement: Supplementary file 1 [file Data_Sheet_1.pdf]

Milk Fat Globules Hamper Adhesion of Enterohemorrhagic *Escherichia coli* to Enterocytes: *In Vitro* and *In Vivo* Evidence

Thomas Douëllou[a,b], Wessam Galia[b], Stéphane Kerangart[b], Thierry Marchal[c], Nadège Milhau[c], Renaud Bastien[d], Marion Bouvier[b, e], Samuel Buff[c], Marie-Christine Montel[a], Delphine Sergentet-Thevenot[b, e]#.

SUPPLEMENTARY DATA

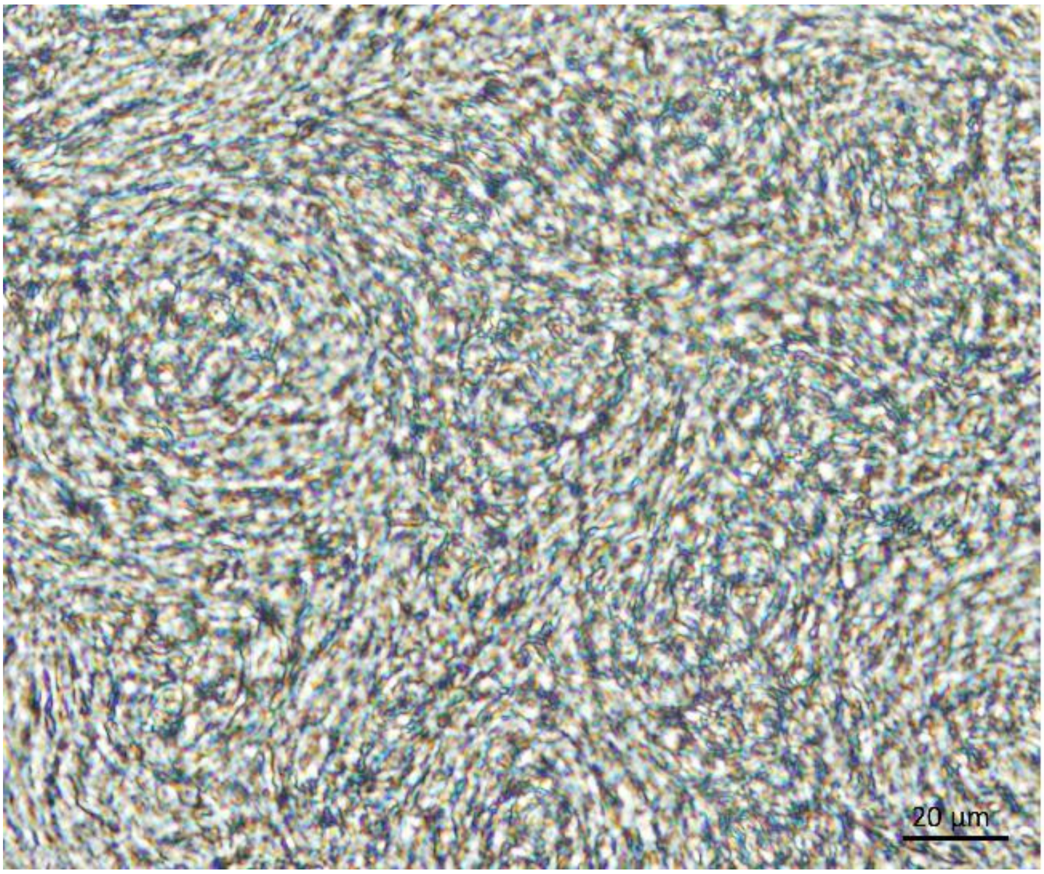

Figure S1: Micrograph of Caco-2/HT-29 MTX monolayer co-cultured *in vitro* negative control for cells monolayer integrity and mucus production verification.

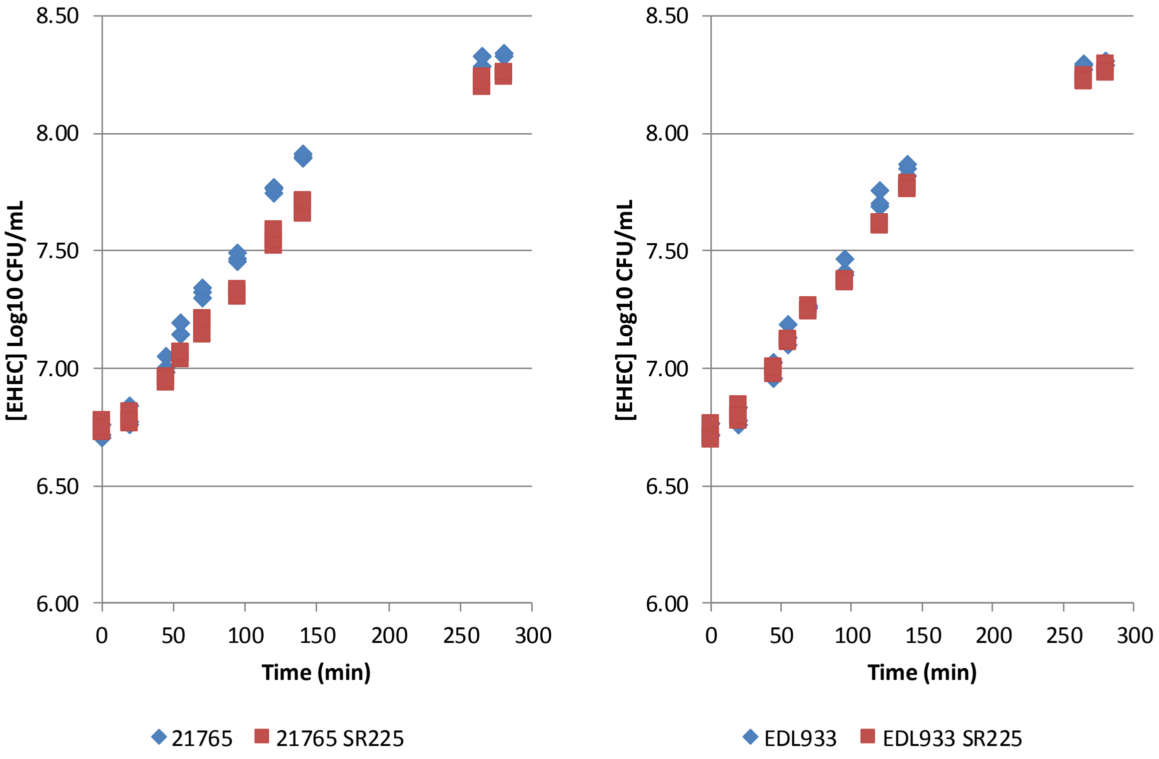

Figure S2: Growth rate of Streptomycin Resistant EHEC strains EDL933 SR225 and 21765 SR225 compared to parental strains.

Microorganism	Selective medium	Incubation
EHEC strains	Coli ID medium + 100 µg/mL of Streptomycin sulfate	1 day, 42 °C
Thermophiles	M17 agar	3 days, 42 °C
Ripening bacteria	Cheese ripening bacteria medium	10 days, 25 °C
Total mesophilic bacteria	Plate count agar + 1% milk	2 days, 30 °C
Gram negative bacteria	Plate count agar + 1% milk + 5 mg/l vancomycin + 5 mg/l crystal violet	2 days, 30 °C
Yeasts/Molds	Oxytetracycline glucose agar	2 days, 25 °C
Pseudomonas	Cetrimid Fucidin Cephalosporin agar	2 days, 25 °C

Table S1: Selective media used to enumerate STEC strains and the main cultivable cheese microflora.

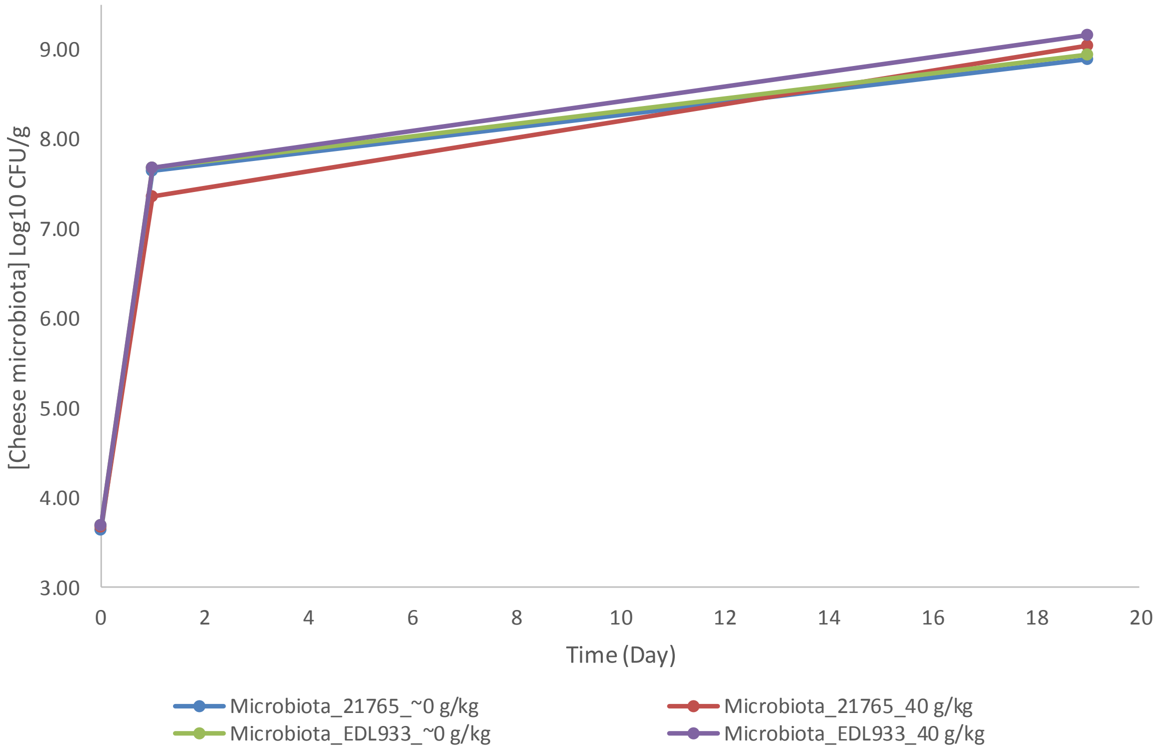

Figure S3: Cheese microbiota evolution in ~0.5% or 40% cheese artificially contaminated with EHEC strain EDL933 SR225 or 21765 SR225.

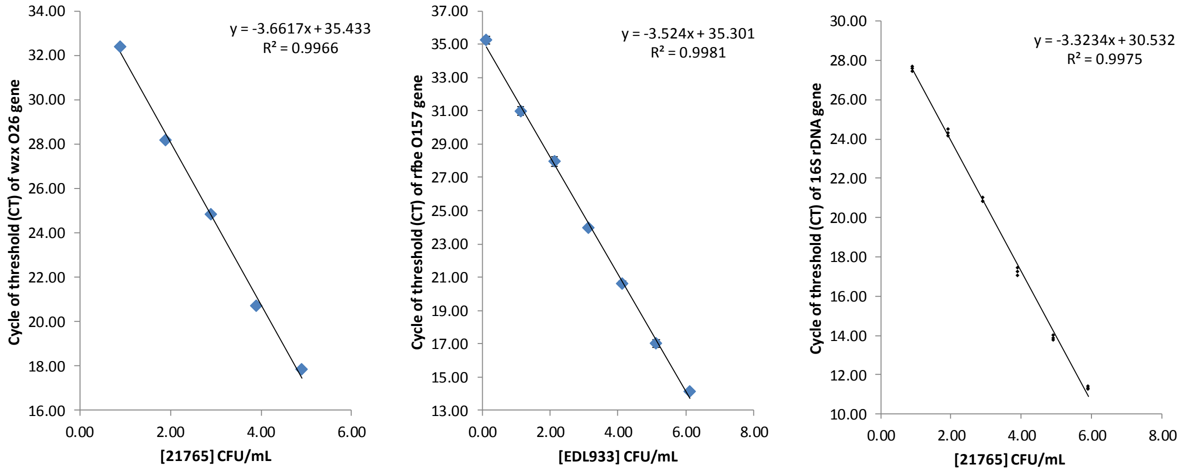

Figure S4: Calculation of the equivalent cells in function of the cycle to threshold (Ct) apparition of O-antigen coding gene (*wzx* O26 or *rfbe* O157) or 16S rDNA gene by rt-qPCR.
